# Wandering spleen torsion—use of contrast-enhanced ultrasound

**DOI:** 10.1259/bjrcr.20150342

**Published:** 2016-06-13

**Authors:** Elisa Aguirre Pascual, Teresa Fontanilla, Íñigo Pérez, Beatriz Muñoz, Maria Soledad Carmona, Javier Minaya

**Affiliations:** ^1^Department of Radiology, Hospital Universitario Doce de Octubre, Madrid, Spain; ^2^Department of Radiology, Hospital Universitario Puerta de Hierro Majadahonda, Madrid, Spain

## Abstract

We report a case of torsion of a wandering spleen in an 18-year-old male patient who presented with acute abdominal pain and left lower quadrant mass. The patient was initially misdiagnosed at another institution. The patient came to our hospital for further investigation. Contrast-enhanced ultrasound was performed and showed a solid hypoechoic avascular mass, which was all that remained of the spleen, located under the left kidney. Based on the ultrasound findings, CT scan and MRI of the abdomen were performed to confirm the suspicion of torsion of a wandering spleen. To the best of our knowledge, there are no case reports describing the use of contrast-enhanced ultrasound for diagnosing torsion of a wandering spleen.

## Clinical presentation and differential diagnosis

An 18-year-old male came to our hospital for further investigation of findings seen on ultrasound and CT scan performed at another institution because of a 12-h history of left flank bulge, nausea and slight left lower quadrant pain. The patient presented to the emergency department because of suspected differential diagnosis of vesical diverticula, mesenteric cyst or lymphangioma inferred from the finding of a very low-attenuation mass on the CT scan.

Physical examination revealed a firm and tender left lower quadrant mass. The patient denied having fever, dysuria or constipation. Laboratory tests revealed decreased platelet count of 135.00 × 10^3^ μl^-1^ (150.0–450.0) and increased white blood cell count of 15.48 × 10^3^ μl^-1^ (4.0–11.0).

## Imaging findings

**Figure 1.**
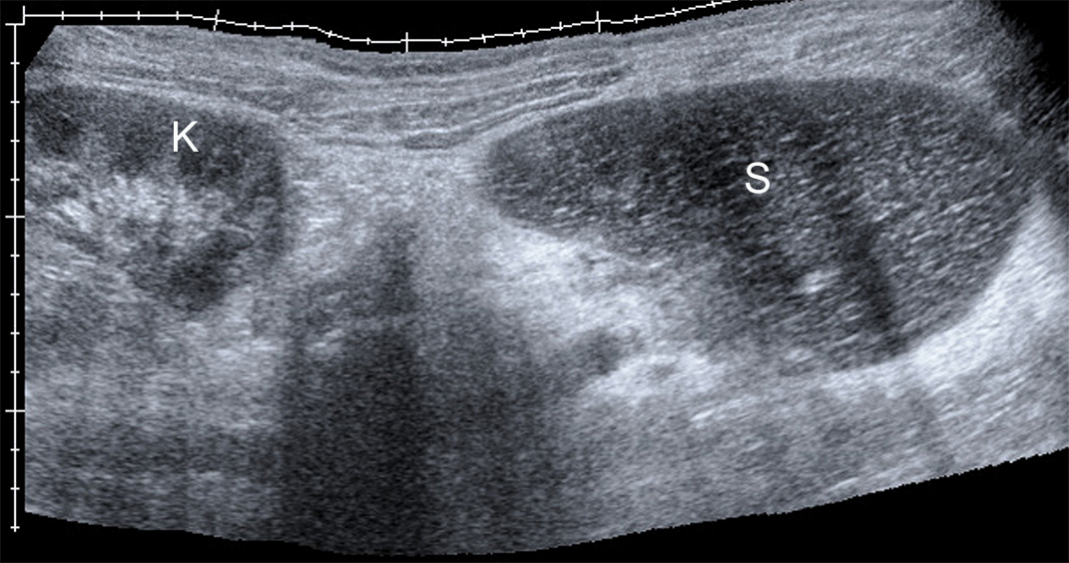
Greyscale ultrasound panorama of the left hypochondrium and flank. The spleen appears as a homogeneous hypoechoic comma-shaped mass (**S**); it should be noted that the spleen is located under the lower pole of the kidney (**K**) in the ectopic position.

**Figure 2.**
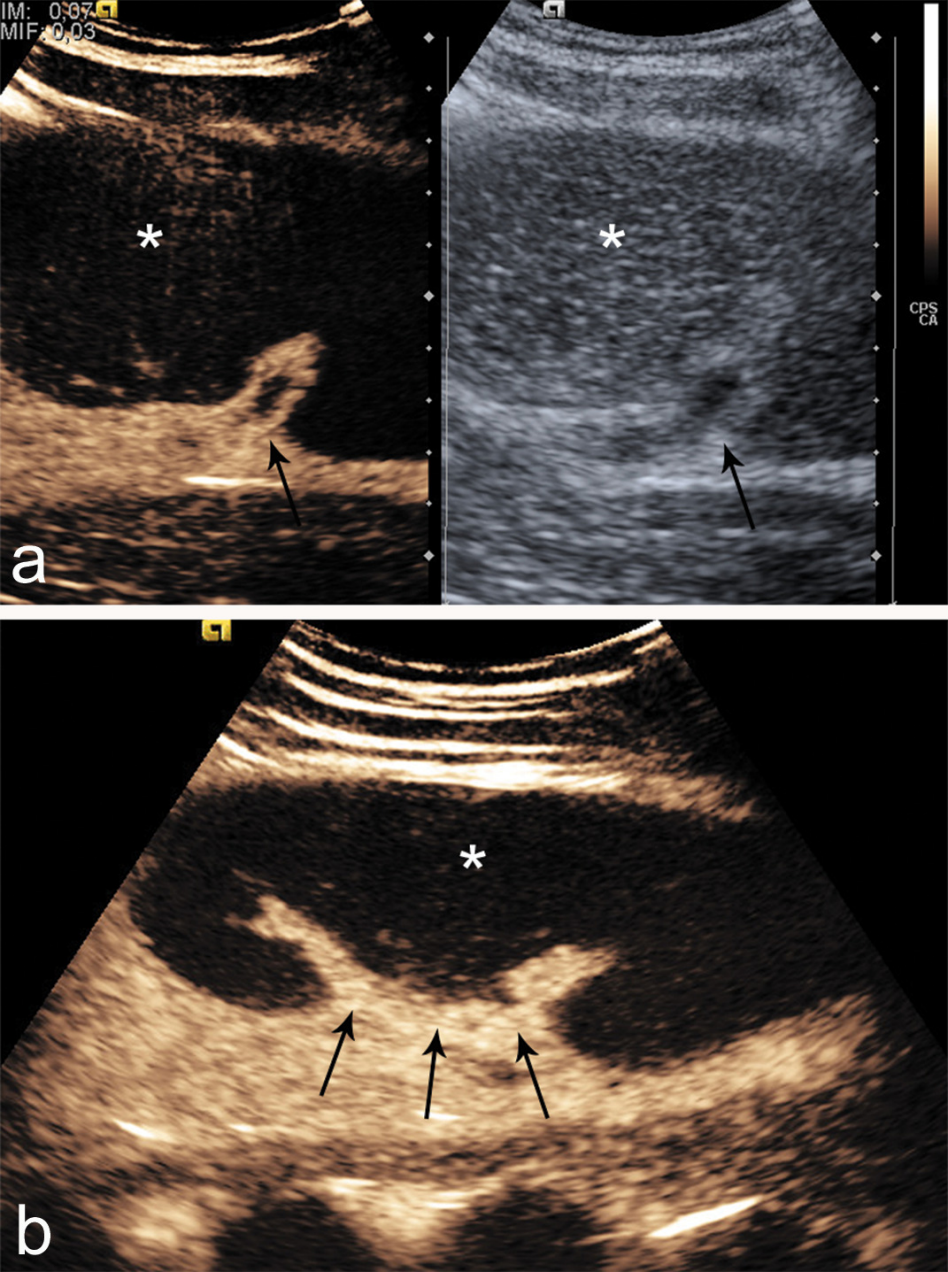
(a) 30 s sagittal contrast-enhanced ultrasound image showing lack of enhancement of the whole organ (asterisks), suggestive of infarction, there is absence of hilum enhancement (arrows). (b) It should be noted that no enhanced arterial or venous vessels are depicted in the splenic hilum (arrows).

**Figure 3.**
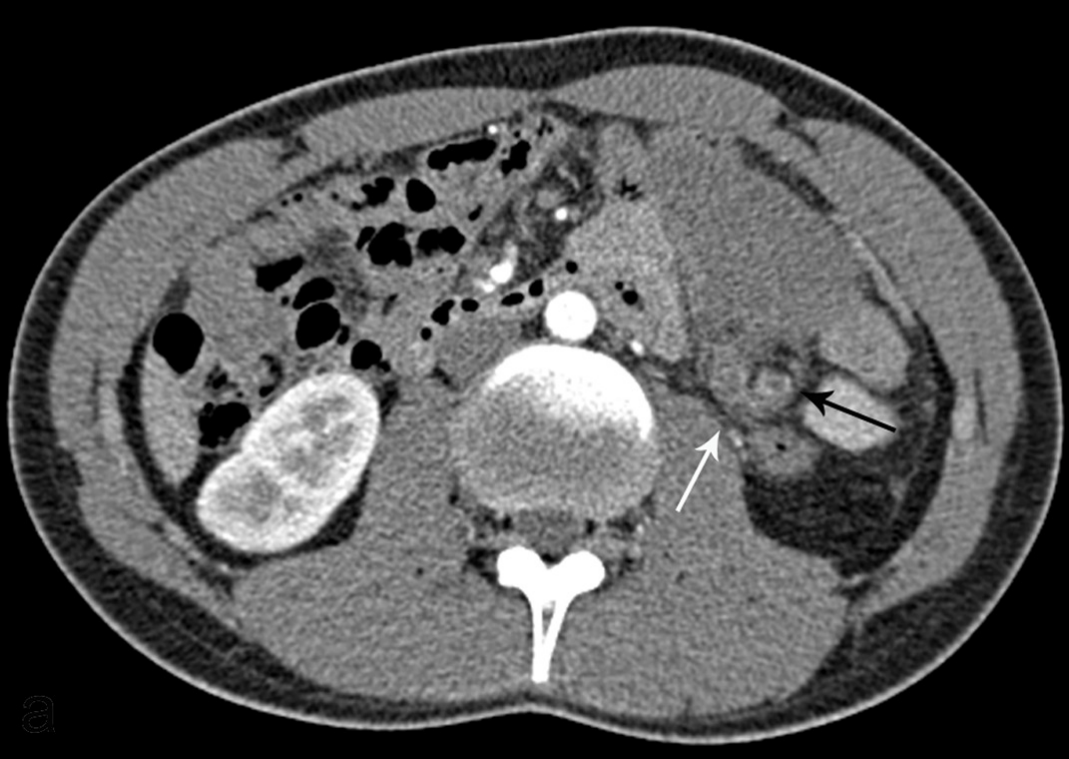
Axial CT arterial phase image shows hypoattenuating left flank mass corresponding to the wandering spleen. The splenic pedicle is twisted (white arrow), giving a whirled appearance that confirms the diagnosis of torsion. The central structure of the whirl is the splenic artery with thrombus inside (black arrow), and the peripheral part corresponds to the vein with thrombus (confirmed in venous phase images, not shown).

**Figure 4.**
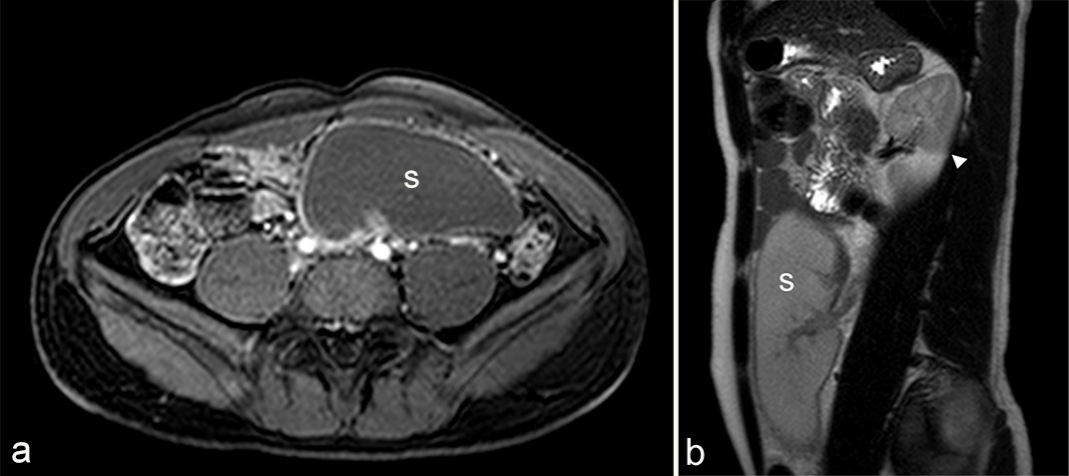
(a) Axial post-contrast *T*_1_ image showing the non-enhancing spleen and thin rim of enhancement of the splenic capsule located in the left lower quadrant (**S**). (b) Axial parasagittal *T*_2_ weighted MRI shows an ectopic spleen located under the left kidney (**S**). Note that the left kidney is slightly malrotated (white arrowhead).

On greyscale ultrasound, the spleen was not seen in the left upper quadrant; instead, there was a 19 cm long solid hypoechoic mass located under the left kidney, which was all that reminded of the spleen (Figure 1). The left kidney was slightly malrotated and without any other anomaly. Doppler ultrasound demonstrated no flow in the splenic parenchyma and hilum. Additional contrast-enhanced ultrasound (CEUS) was performed after intravenous administration of 2.4 ml sulphur hexafluoride-filled microbubble, a second-generation ultrasound contrast agent (Sonovue; Bracco, Milan, Italy). CEUS showed lack of enhancement of the whole mass and no enhancement of the vessels at the hilum (Figure 2). Based on the ultrasound findings, CT scan and MRI of the abdomen were performed to confirm the suspicion of torsion of a wandering spleen with infarction. The CT scan depicted an enlarged ectopic comma-shaped spleen located under the left kidney, diffusely hypoattenuating without enhancement. Axial CT imaging showed whorled appearance of a twisted splenic pedicle,which confirmed the diagnosis of torsion (Figure 3). MRI scan showed diffuse hypointense *T*_1_ and hyperintense *T*_2_ weighted images of the spleen without enhancement, which was consistent with infarction in the whole parenchyma (Figure 4). The splenic pedicle appeared twisted, giving it a whirled appearance, which confirmed the diagnosis.

## Treatment and outcome

Owing to the suspected diagnosis of torsion of a wandering spleen, laparoscopic splenectomy was performed, which confirmed the imaging findings. The patient had an uneventful recovery.

## Discussion

Wandering spleen is an unusual entity (incidence < 0.5%) in which a long pedicle allows the spleen to migrate from the normal splenocolic angle to the lower abdominal cavity. The spleen is held in position by three ligaments—gastrosplenic, splenorenal and phrenicocolic.^1^ When these ligaments are excessively lax or maldeveloped, the spleen may acquire an abnormal position. There are acquired risk factors associated with this pathology such as splenomegaly, pregnancy and trauma.^1^

Generally, patients with wandering spleen are asymptomatic, but some patients may present with a palpable abdominal mass. Torsion is the main complication, which may lead to ischaemia and infarction, or even splenic rupture; thus prompt detection would benefit patients with this condition.

Clinical diagnosis is difficult owing to non-specific symptoms ranging from being asymptomatic to abdominal pain. Diagnosis is based on imaging findings; various imaging modalities can be used. In most cases, ultrasound is usually the first imaging technique used for making a diagnosis. The normal spleen parenchyma is very homogeneous and is more echogenic than the liver and left kidney. A wandering spleen is not seen in the upper abdomen, but is visible in the left lower quadrant or pelvis on radiological evaluation. Infarcts may be difficult to visualize on greyscale ultrasound owing to their variable appearance, depending on the timing and grade of torsion.^2^ Usually they are hypoechoic and wedge shaped, but they may be isoechoic, especially if the torsion is recent. Colour Doppler and power Doppler show areas of signal absence, which suggests perfusion defects, but Doppler ultrasound may be suboptimal in some patients; CEUS detects the smallest microvessels and obtains a perfusion map of the organs. CEUS has been shown to be a safe^3^ and appropriate additional tool for depicting perfusion of the splenic parenchyma and detecting repletion vascular defects.^4–6^ The microbubble contrast increases the sensitivity and specificity of the exploration, improving the capacity to diagnose splenic infarcts.^5,6^ Typically, infarcts are seen as non-enhanced areas, often as wedge shaped with the wide base orientated to the spleen surface. The border is usually well defined, although there may be a fuzzy border owing to partially ischaemic peripheral areas (watershed areas). If the infarct is complete, there is a complete absence of spleen enhancement. Often, a peripheral rim enhancement is seen on CEUS, as seen around infarcted organs, for example, the kidney.

CT scan and MRI confirm the suspicion of torsion of a wandering spleen, showing its ectopic position. Typically, the splenic hilum appears twisted; the whirled appearance is a very specific sign of torsion of the splenic pedicle.^7^ Lack of parenchymal and rim enhancement of the splenic capsule is a typical finding of post-contrast imaging.

Pre-operative diagnosis is based on radiological findings, but these are not always unequivocal; for instance, our case was initially misdiagnosed. Hence awareness of this entity allows early diagnosis and appropriate management.^8,9^ Depending on the grade of pedicle torsion, treatment can be detorsion and splenopexy, or splenectomy.^10^ Laparoscopic splenopexy is preferred if possible, especially in patients ≤ 30 years of age, owing to eventual infectious complications. Splenectomy is the treatment of choice in case of infarction. To the best of our knowledge, there are no case reports describing the use of CEUS in diagnosing torsion of a wandering spleen.

## Conclusions

Wandering spleen is a rare entity; its main complication is torsion, which can be challenging to diagnose owing to non-specific symptoms. The first imaging technique for making diagnosis is ultrasound, additional CEUSincreases sensitivity to allow diagnosis of vascular patency and parenchymal viability. Recognition of wandering spleen and its complications is of utmost importance for deciding on proper surgical treatment options.

## Learning points

1. Wandering spleen torsion is a rare cause of acute abdomen with significant morbidity and mortality if misdiagnosed.
2. CEUS is a rapid and sensitive technique for diagnosing infarction with certainty and has an impact on patient management.
3. Treatment options are splenopexy or splenectomy, depending on the grade of torsion of the pedicle.

## Consent

Written informed consent for the case to be published (including images, case history and data) was obtained from the patient.
